# Effects of Dietary Nutrients on Fatty Liver Disease Associated With Metabolic Dysfunction (MAFLD): Based on the Intestinal-Hepatic Axis

**DOI:** 10.3389/fnut.2022.906511

**Published:** 2022-06-17

**Authors:** Nan Yao, Yixue Yang, Xiaotong Li, Yuxiang Wang, Ruirui Guo, Xuhan Wang, Jing Li, Zechun Xie, Bo Li, Weiwei Cui

**Affiliations:** ^1^Department of Epidemiology and Biostatistics, School of Public Health, Jilin University, Changchun, China; ^2^Department of Nutrition and Food Hygiene, School of Public Health, Jilin University, Changchun, China

**Keywords:** fatty liver disease associated with metabolic dysfunction, MAFLD, dietary nutrients, intestinal-hepatic axis, non-alcoholic fatty liver disease, NAFLD

## Abstract

Non-alcoholic fatty liver disease (NAFLD) has recently become the most common liver disease with a global prevalence of over 25% and is expected to increase. Recently, experts have reached a consensus that “fatty liver disease associated with metabolic dysfunction or MAFLD” may be a more appropriate and inclusive definition than NAFLD. Like the former name NAFLD, MAFLD, as a manifestation of multiple system metabolic disorders involving the liver, has certain heterogeneity in its pathogenesis, clinical manifestations, pathological changes and natural outcomes. We found that there is a delicate dynamic balance among intestinal microflora, metabolites and host immune system to maintain a healthy intestinal environment and host health. On the contrary, this imbalance is related to diseases such as MAFLD. However, there are no clear studies on how dietary nutrients affect the intestinal environment and participate in the pathogenesis of MAFLD. This review summarizes the interactions among dietary nutrients, intestinal microbiota and MAFLD in an attempt to provide evidence for the use of dietary supplements to regulate liver function in patients with MAFLD. These dietary nutrients influence the development and progression of MAFLD mainly through the hepatic-intestinal axis by altering dietary energy absorption, regulating bile acid metabolism, changing intestinal permeability and producing ethanol. Meanwhile, the nutrients have the ability to combat MAFLD in terms of enriching abundance of intestinal microbiota, reducing *Firmicutes/Bacteroidetes* ratio and promoting abundance of beneficial gut microbes. Therefore, family therapy with MAFLD using a reasonable diet could be considered.

## Introduction

Since ancient times, oral feeding has been an important way for human survival. Humans obtain various nutrients required for life through food consumption, which consists of carbohydrates, lipids, amino acids, dietary fiber, minerals, and vitamins. In addition to the common nutrients, there are other dietary components such as coffee (rich in caffeine) and tea (containing tea polyphenols) which are an integral part of our daily diet. In this review, nutrients and dietary components are collectively referred to as dietary nutrients.

The intestinal tract is the largest digestive organ of the human body and is called the second brain of the human body. As the intestine contains a variety of digestive juices, it is the main place for the absorption of various dietary nutrients (1). Besides, the gut microbiota is an important component in maintaining healthy homeostasis in the intestine. The strains of the gut microbiota vary widely from individual to individual and are strongly influenced by the host genotype, initial colonization by vertical transmission at birth and dietary habits (2–5). In general, there is a delicate dynamic balance between the gut microbiota, metabolites, and the host immune system to maintain a healthy intestinal environment and host health (6). Conversely, this imbalance has been associated with disease ranging from localized gastrointestinal disorders to neurological, respiratory, metabolic, and cardiovascular diseases (7).

Non-alcoholic fatty liver disease (NAFLD) has recently become the most common liver disease with a global prevalence of over 25% (8) and is expected to increase (9). It includes simple steatosis (NAFLD) and non-alcoholic steatohepatitis (NASH) (10), and is characterized by excessive intracellular fat deposition in the liver, which does not include alcohol and other well-defined hepatic impairment factors. There is a bidirectional material exchange pathway (intestine-liver axis) between the liver and the intestine (11), so the occurrence and development of NAFLD may be closely related to intestinal microorganisms.

Recently, experts have reached a consensus that “fatty liver disease associated with metabolic dysfunction or MAFLD” may be a more appropriate and inclusive definition than NAFLD (12). Like the former name NAFLD, MAFLD, as a manifestation of multiple system metabolic disorders involving the liver, has certain heterogeneity in its pathogenesis, clinical manifestations, pathological changes and natural outcomes. MAFLD is diagnosed on the basis of evidence of hepatic steatosis (by imaging, blood biomarkers, or hepatic histology) associated with any one or more of the following: evidence of overweight/obesity, T2DM, metabolic disorders (13). Recent studies have shown a higher global prevalence of MAFLD than NAFLD (14). A study of a representative sample of the general population in the United States found that the prevalence of MAFLD in the United States from 2017 to 2018 was 39.1%, while approximately 7.4% of patients with MAFLD had advanced hepatic fibrosis (15). In China, the prevalence of MAFLD in adults 40 years of age and older is estimated at 40.3% and high risk of advanced fibrosis based on fibrosis-4 was highly prevalent (14.7%) in lean MAFLD with T2DM (16). In conclusion, MAFLD is a major factor affecting health and would evolve into a serious public health problem.

However, there are no clear studies on how dietary nutrients affect the intestinal environment and participate in the pathogenesis of MAFLD. This review summarizes the interactions among dietary nutrients, intestinal microbiota and MAFLD in an attempt to provide evidence for the use of dietary supplements to regulate liver function in patients with MAFLD.

## Healthy Gut Microbiota: Composition and Function

The gastrointestinal microenvironment plays a central role in maintaining homeostasis of healthy host, consisting of monolayer cell epithelium, a local immune system, and the microbiome (17). Among them, the intestinal epithelium has the function of absorbing nutrients, resisting invading microorganisms in the intestinal lumen (through physical and chemical means) and providing a semi-permeable barrier between the host and the intestinal lumen (17). Besides, as more than 80% of the body's lymphocytes are found in the gut, the microbiome forms a local intestinal immune system with both surveillance and effector arm functions (18). Most importantly, the gut microbes, known as the “second brain” of the body, are the third component of the intestinal microenvironment. It is involved in the composition of the intestinal microenvironment and in regulating the dynamic balance of it.

The human gut microbiome is a complex ecosystem of bacteria, yeasts and viruses that regulate the interactions between the human host and its environment (19). There are more than 1,000 species of bacteria in the intestine, 90% of which are from *Firmicutes* (mainly composed of Gram-positive *Clostridia*) and *Bacteroidetes* (mainly composed of Gram-negative bacteria such as *Bacteroides* fragilis) (20). In addition, *Actinobacteria, Proteobacteria, Fusobacteria* and *Verrucomicrobia* are also the dominant microbial phyla of the intestine (21).

These enormous numbers of bacteria can be broadly divided into three broad categories: beneficial, pathogenic and neutral bacteria. Beneficial bacteria, also known as probiotics, are indispensable for human health and include various species of *Bifidobacteria* and *Lactobacilli* (22). They are involved in the metabolism of nutrients, promotion of intestinal motility, inhibition of the growth of pathogenic bacteria and decomposition of pathogenic and toxic substances. Table 1 lists the microorganisms commonly used as probiotics (23–26).

**Table 1:** Microorganisms used as probiotics.

**Genus**	**Species**
**Bacteria**	**Lactobacillus species**
	L.acidophilus
	L.bulgaricus
	L.casei
	L.crispatus
	L.fermentum
	L.gasseri
	L.johnsonii
	L.lactis
	L.plantarum
	L.reuteri
	L.rhamnosus GG
	**Bifdobacterium species**
	B.adolescentis
	B.animalis
	B.bifidum
	B.breve
	B.infantis
	B.lactis
	B.longum
	**Bacillus cereus**
	**Enterococcus faecalis**
	**Enterococcus faecium**
	**Escherichia coli Nissle**
	**Streptococcus thermophilus**
**Yeast**	**Accharomyces boulardii**

The gut microbiome has multiple functions and supports the balance of the intestinal microenvironment. Intestinal microbes can symbiotically interact with the intestinal barrier and influence its permeability (27). Intestinal permeability is an essential marker of intestinal barrier function, which is tightly regulated in homeostasis and is closely associated with disease. The gut microbiome may act directly on intestinal permeability by influencing tight junctions (TJ) characteristics and activity, and indirectly by modulating inflammation (28). In addition, gut microbes may influence the immune response in the evolving tumor microenvironment by triggering a pro-inflammatory or immunosuppressive program, ultimately intervening in tumourigenesis and progression.

**Figure 1:**
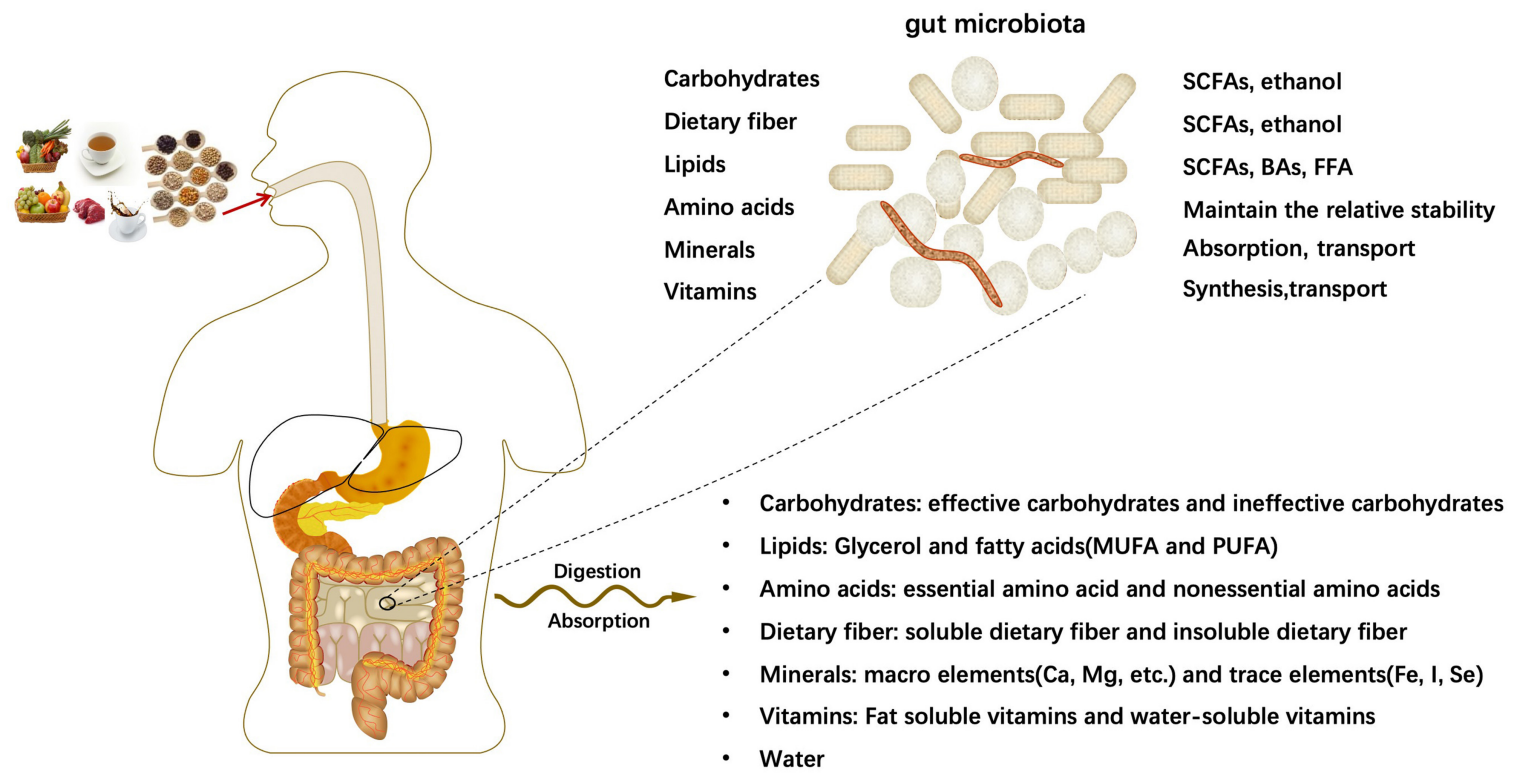
Digestion and absorption of nutrients.

The human body takes in a variety of meals daily to meet various growth and development needs of the body. After being digested and absorbed by the digestive system, all kinds of meals will mainly provide major nutrients for the human body: carbohydrates, lipids, amino acids, dietary fibers, minerals, vitamins and other dietary components. The intestine is the main place for digestion, and the microflora parasitic on the human intestine has the function of processing nutrients. With the participation of intestinal microflora, the relative homeostasis of nutrients in the host is maintained. Intestinal microorganisms further decompose nutrients into smaller units, such as bile acids(BAs) (29), short-chain fatty acids(SCFAs) (30, 31), free fatty acid (FFA), which contribute to the transport and absorption of nutrients. In some cases, intestinal microorganisms generate new substances (Figure 1).

## Dietary Nutrients Affect the Intestinal Microenvironment and Gut Microbiota

Dietary nutrients enter the digestive system for digestion and absorption and are transformed into small molecule metabolites such as SCFAs and BAs and produce a range of biological effects. This process may affect the intestinal microenvironment and intestinal microbiota by altering dietary energy absorption, regulating BAs metabolism, changing intestinal permeability and producing ethanol.

### Alter Dietary Energy Absorption

#### Carbohydrates

As a breakdown product of carbohydrates, the metabolism of SCFAs is influenced by the type and number of microorganisms in the intestinal tract (32), and is a regulator of pH, cell volume and other functions related to ion transport, epithelial cell nutrition, and a regulator of proliferation, differentiation and gene expression (33). SCFAs are beneficial to the health of the host's intestinal environment, as they indirectly affect the microbial communities by reducing the pH of the intestinal environment (when the pH of the intestinal lumen is acidic, the number of potentially pathogenic *Clostridium* perfringens is reduced) (34), which contributes to the health of the host.

In addition, dietary interventions targeting people at high risk of metabolic syndrome have demonstrated that high-carbohydrate diets modulate glycolytic bacteria in human feces, including *Bacillus mimicus* and *Bifidobacterium bifidum*, both of which are independently associated with improved body energy regulation and reduced risk factors for metabolic syndrome (35).

#### Lipids

The olive oil diet enhances microbial metabolism of SCFAs, increases ketone body synthesis and degradation, and strengthens organism immunity, whereas the corn oil diet is closely associated with lipid metabolism and carbohydrate metabolism (36). Both the corn oil diet and the milk fat diet enhance carbohydrate metabolism, which may be an indirect pathway for the action of lipids on the gut microbiota (36).

#### Amino Acids

Konomi et al. used a high fat diet (HFD) with casein and an HFD with soy protein to intervene in 105 ICR 8-week-old male mice. The pH value in the intestinal lumen of mice in the soy group decreased and the content of SCFAs increased, showing that the levels of acetic acid, propionic acid, lactic acid and butyric acid tended to be significantly higher or higher than those in the casein group (37).

#### Dietary Fiber

The benefits of dietary fiber on the intestinal flora are dependent on the action of SCFAs, the enzymatic product of soluble dietary fiber. SCFAs lower the colonic pH below the threshold for pathogenic bacteria, thereby inhibiting the growth of potential pathogens and promoting the cultivation of beneficial bacteria such as *Bifidobacteria* and *Lactobacilli* (38, 39). Meanwhile, SCFAs promotes the strengthening of the intestinal barrier function, thus reducing the infestation of pathogenic bacteria and hazardous substances to the host (40). Evidence for this is the finding that application of a mixture of SCFAs alone and in combination raised trans-epithelial resistance and reduced paracellular transport markers in the rat cecum wall (41).

#### Minerals

Limited studies have indicated that phosphorus supplementation affects SCFA and gut microbial diversity. The experimental group supplemented with 1000 mg/day of phosphate showed significantly higher concentrations of total SCFAs and acetate, and improved gut microbiome diversity after 8 weeks (42). In addition, elevated abundance of butyrate-producing *Faecalibacterium* and *Pseudoflavonifractor* was detected in cecum samples from broilers whose diets were supplemented with phosphorus (43).

#### Other Dietary Components

After entering the body, coffee is metabolized by the gut microbiota into fermentation products such as SCFAs (44, 45). In an animal experiment, mice with metabolic syndrome fed with chlorogenic acid and caffeine (the main component of coffee) had a 1.5- to 4-fold increase in propionate and a 1.5- to 3-fold increase in butyrate, in addition to restoring the already reduced levels of acetate in mice with metabolic syndrome compared to controls (46). Meanwhile, Sergio et.al extracted mannooligosaccharides from spent coffee grounds and submitted them to an *in vitro* fermentation with human feces. They observed that SCFAs production increased in a dose-dependent manner (44).

### Regulate BAs Metabolism

BAs are an essential part of the molecular environment of the healthy human gut (47) and are synthesized from cholesterol mainly in the hepatocytes and then transported to the gallbladder for storage. Ingestion of the diet will stimulate the excretion of BAs into the duodenum, of which 95% will be reabsorbed in the intestine and returned to the liver via the enterohepatic circulation, while the remaining 5% is excreted in the feces (48, 49). BA functions as a digestive activator, facilitating the dissolution and digestion of lipophilic exogenous substances, fat-soluble vitamins, fatty acids and glycerol monoesters (50).

The rationale is that amino acid intervention increases *Clostridium* perfringens cluster XIVa, which has the ability to promote the production of secondary BAs from primary BAs, in the feces of mice and can influence the metabolic process of BAs (51). In addition, it was suggested that the microbiota may contribute to changes in BA translocation and circulating BA in the gut, and that BAs and gut microbes interact to influence each other's abundance and size (52).

BAs use the immune system to remodel the intestinal flora through farnesoid X receptor(FXR) mediation (53, 54), while the intestine-hepatic FXR-FGF(fibroblast growth factor)15-FGFR4 signaling axis can regulate the relationship between BAs metabolism and intestinal microbes (55). Bile salt hydrolase (BSH) is an important substance in the metabolism of BAs, and it has been suggested that BSH may be an important link in the effect of BAs on intestinal flora (55). Studies have shown that theaflavins inhibit BSH-associated microorganisms and BSH activity, which leads to increased BA binding in the ileum, further inhibiting the intestinal FXR-FGF15/19 signaling pathway and enhancing hepatic BA production (55), ultimately affecting the number and composition of intestinal microorganisms.

### Change Intestinal Permeability

The intestinal barrier is the functional entity that separates the intestinal lumen from the internal host and consists of mechanical components (mucus, epithelium), humoral components (defensins, IgA), immune components (lymphocytes, innate immune cells), muscles and neuronal components. Intestinal permeability is understood to be a measurable characteristic of the intestinal barrier. Impaired intestinal permeability, on the other hand, implies a non-transitory alteration of permeability disturbance, leading to loss of intestinal homeostasis, dysfunction and disease (56).

Intestinal permeability could be regulated by prebiotics, probiotics and diet. SCFAs are organic acids of propionate, butyrate and valerate, which are produced by the fermentation of intestinal microorganisms from undigested dietary carbohydrates in the colon. In particular, butyrate plays a particular role in maintaining the intestinal barrier. In inflammatory bowel disease (IBD), a persistent state of inflammation will lead to leakage of the TJs of the patient's intestine, and butyrate enemas help to reduce intestinal inflammation in patients (57), while preserving TJ integrity and directly improving intestinal barrier function by inhibiting the release of TNF-α, IL-13, etc. (58–60). Although some studies have reported denying the role of intestinal flora in maintaining the integrity of the intestinal barrier, there are numerous studies confirming it (61, 62). For example, the probiotic *E. coli* Nissle 1917 (EcN) has been shown to prevent barrier disruption caused by intestinal pathogenic *E. coli* strains infecting T84 and Caco-2 cells (63). Metabolites secreted by Bifidobacterium infantis Y1 (one of the components of the probiotic product VSL#3) lead to increased expression of ZO-1 and occludin, while decreasing the expression of claudin-2, thereby enhancing the effect on trans-epithelial resistance and ion secretion (64). Both high-fat diets and Western-style diets characterized by high carbohydrate and high-fat diets are thought to enhance intestinal permeability and cause metabolic endotoxemia (65–67). The effect of diet on intestinal permeability is largely dependent on the host's intestinal microbiota and genetic susceptibility. 4-week-old C57Bl/6 male mice fed a high-fat diet for 3 months found that increased intestinal permeability in the ileum and cecum of diabetic mice promoted the effects of specific microbiota and resulted in enhanced endotoxaemia (67). Oligofructose has been shown to promote selective microbiota changes (*Bifidobacterium*) leading to increased endogenous glucagon-like peptide 2 (GLP-2) production, thereby improving intestinal barrier function and providing tighter junctions and less inflammation (68).

#### Carbohydrates

Easily digestible carbohydrates (e.g., sucrose, fructose, glucose, maltodextrin and corn starch) and indigestible carbohydrates (i.e., fibers such as, cellulose, methylcellulose, psyllium, pectin, inulin, linseed, marshmallow root, potato starch and slippery elm) have an effect on the density of intestinal flora, thereby altering intestinal permeability and affecting the degree of colonic inflammation in mice (69).

#### Lipids

The effects of saturated and unsaturated fatty acids on intestinal endotoxin transport and postprandial endotoxaemia in pigs were assessed by Venkatesh et al. who found that coconut oil (high in saturated fatty acids) increased intestinal permeability, whereas cod liver oil and fish oil (very high in monounsaturated oleic acid) reduced intestinal permeability (70) (very high in monounsaturated oleic acid) reduced intestinal permeability, suggesting that dietary oils may differentially alter intestinal endotoxin transport through the regulation of intestinal membrane permeability by fatty acids (70).

#### Amino Acids

Konomi et al. found that the alpha diversity was significantly higher in the soya group than in the casein group, and significantly higher in the *Bacteroides, Proteus, Bifidobacterium, Enterococcus, Vibrio vulnificus* and *Vibrio desulfuricans* than in the casein group. Meanwhile the thick-walled phylum was significantly lower than in the casein group, with *Vibrio desulfuricans* being associated with intestinal barrier dysfunction (71).

#### Minerals

In a 54-day nutritional intervention, high calcium supplementation (12 g/kg) modulated the intestinal microbiota in a high-fat diet mouse model, as evidenced by an increase in the number of *Bifidobacteria* and the *Bacteroidetes/Probacteria* ratio in cecum samples (72). The mechanism could be that dietary calcium reduces the cytotoxicity of intestinal contents and intestinal epithelial cell lysis by precipitating cytotoxic surfactants (e.g., BAs). The reduced tubular cytotoxicity not only enhances the barrier function of the intestinal mucosa but also the protective endogenous microbial community (73). Zinc(Zn) is a mineral associated with the maintenance of the mucosal barrier and is essential in the maintenance of intestinal homeostasis (74). Shigella infection was reported to cause significant phosphorylation of extracellular signal-regulated kinase (ERK), leading to barrier disruption, while Zn^2+^ reversed this ERK activation and enhanced barrier integrity (75). Animal studies show that zinc-amino acid complexes diminish the pathological changes in intestinal permeability caused by Clostridium perfringens in broilers (76).

#### Vitamins

Vitamin D contributes to maintaining TJs to safeguard the integrity and function of the intestinal barrier. It has been suggested that vitamin D3/VDR(vitamin D receptor) signaling could regulate the number and distribution of tight junction proteins (77, 78). Simultaneously, vitamin D supplementation in the presence of a functional VDR strengthens the epithelial barrier by reducing the paracellular permeability of polarized epithelial cells (79, 80).

Vitamin A also affects the metabolism of SCFAs, as evidenced by significantly higher levels of butyrate and acetate and bacterial genes associated with butyrate production (but and buk) in the cecum of A+ mice compared to A- mice (81). It is worth noting that retinoic acid (RA, a metabolite of vitamin A) induces the expression of IL-22 binding protein in dendritic cells and promotes intestinal homeostasis (82). IL-22 is a cytokine involved in the homeostasis and repair of intestinal barrier function and affected the permeability associated with the epithelial TJs of claudin-2 (83).

Vitamin C has a similar role in maintaining the barrier function of the intestinal tract. Dietary vitamin C supplementation decreased intestinal barrier defects in alcohol-fed guinea pigs as observed in an animal study (84). A possible reason for increasing vitamin C intake to improve intestinal barrier function is the ability of vitamin C to increase collagen synthesis in the intestine (85). This proposed mechanism is consistent with the vitamin C's coenzyme function of hydroxylating proline and lysine to cross-link collagen (86).

### Produce Ethanol

Endogenous ethanol is usually derived from the breakdown of carbohydrates by intestinal bacteria and is metabolized in the liver by the enzyme ethanol dehydrogenase. Various diets are associated with high levels of ethanol-producing bacterial strains [e.g., *Escherichia coli, Bacteroides*, and *Clostridium* (87–89)] in the intestine. The number of *Bacteroides* in the cecum of vitamin A-deficient (A-) mice was significantly lower than that of vitamin A-sufficient (A+) mice (81). Coffee decreases the amount of *Clostridium* and *Escherichia* coli (90, 91). The abundance of *Clostridium* is closely related to fat intake and can be reduced by the consumption of almond (36) and soybean oil (92).

## Interaction of Gut Microbiota With MAFLD

### Definition and Potential Mechanisms of MAFLD

MAFLD is a clinicopathological syndrome characterized by excessive fat deposition in hepatocytes (10). Patients with MAFLD suffer from hepatocellular damage, inflammation and fibrosis—significant risk factors for the development of cirrhosis and hepatocellular carcinoma (HCC) (93). This means that a certain percentage of MAFLD patients may develop cirrhosis and hepatocellular carcinoma, posing a serious risk to human life and health (94).

However, the potential mechanisms of MAFLD occurrence and progression have not been fully elucidated. It is generally accepted that MAFLD is the result of a combination of multiple factors such as diet, genetics, environment, overweight or obesity, hormones secreted by adipose tissue (leptin, adiponectin), and crosstalk between different organs or tissues (95).

### MAFLD Patients May Have Gut Microbial Disorders

Several studies have indicated that patients with MAFLD often suffer from dysregulated gut flora to some degree. The gut microbiota of patients with MAFLD is characterized by a low abundance of microorganisms, an increase in *Firmicutes/Bacteroidetes* ratio, and a decrease in the abundance of certain bacteria.

An observational case-control study using multi-label pyrophosphate sequencing to determine fecal microbial characterization in patients with suspected MAFLD and healthy subjects found a significant reduction in gut microbiota abundance in patients with MAFLD (96). Among 73 obese children and adolescents in the pediatric clinic, subjects with MAFLD had significantly lower bacterial alpha-diversity than those with simpleobesity (97). A prospective cross-sectional study showed that the relative abundance of anthropoid bacteria in NASH was low and non-related to BMI and energy intake from dietary fat (98).

The *F/B* ratio is a marker of ecological dysregulation associated with a variety of metabolic diseases (99). Ayesha et al. observed that subjects with MAFLD showed a higher *F/B* ratio compared with those without MAFLD (97). Increased *F/B* ratios are commonly found in patients from MAFLD with comorbid obesity. In a cross-sectional study from Indonesia, researchers stratified MAFLD patients by BMI and found a moderate positive correlation between the *Thick-walled phylum/Bacteroid phylum* ratio and steatosis in the obese group (r = 0.435; *P* = 0.030) (100).

There is a delicate dynamic balance between microorganisms in the human gut, with beneficial bacteria being the dominant force in a healthy intestinal microenvironment. When the balance is disturbed by factors such as inflammation, the abundance and ratio of various microorganisms will become disordered. Contrary to expectations, elevated abundance of *Lactobacillus*, which is often used as a probiotic, is generally observed in patiets with MAFLD. Jiang et al. measured the fecal microbiota of histologically confirmed MAFLD patients and healthy controls and found that the abundance of *Lactobacillus* was increased in MAFLD patients (101). The same results were observed in other studies by Da Silva et al. (102) and Raman et al. (96). In a prospective cross-sectional study, 39 adults with biopsy-proven MAFLD and 28 healthy controls were evaluated for gut microbiome and the researchers found lower *Coprococcus* abundance in patients with MAFLD (102). *Coprococcus* is a microorganism involved in the metabolism of alcohol and the pathogenesis of MAFLD (103). Furthermore*, Escherichia* is an endogenous alcohol-producing bacterium that is inextricably linked to MAFLD, as evidenced by the increased abundance of *Escherichia* in patients with MAFLD (87, 101, 104–106).

### Gut Microbes Could Influence the Development of MAFLD

Microbiota can ameliorate or exacerbate MAFLD through a variety of mechanisms. However, the relationship between the factors and the development or progression of MAFLD remains controversial. These parameters are briefly described here.

Firstly, gut microbes may influence energy extraction, absorption, utilization and storage, and these processes may as well be involved in the pathogenesis of MAFLD. Studies have reported that gut flora may increase the absorption of SCFAs, FFA and carbohydrates. Furthermore, it upregulates ChREBP and SREBP-1c and inhibits fating induced adipose factor (Fiaf), leading to the activation of lipoprotein lipase inhibitors (LPL) (107), ultimately leading to increased adipogenesis and the development of a pathological environment for MAFLD (108).

Secondly, intestinal microorganisms have the ability to reduce the levels of choline, converting it into toxic methylamine which is associated with the risk of inducing hepatic steatosis (109). In addition, BAs are another substance that gut microbes influence the pathogenesis of MAFLD. Not only does BAs contribute to lipid absorption and transport, but they are increasingly being considered as nuclear receptor binding agents and play a putative role in altering the microbiome. Experimental animal studies show that treatment of MAFLD mouse models with dual FXR/TGR5(Takeda G protein-coupled receptor 5) bile acid receptor agonists improves disease and alters the phenotype of the intrahepatic macrophage population (110). In another animal study, treatment with antibiotics or the potent antioxidant tempol altered the gut microbiota in mice with increased levels of bound BAs compared to controls (111). Simultaneously, after a high-fat diet was given to both the control and treatment groups, the treatment group had an increase in bound BA metabolites (tauro-β-rhodopsin) and inhibited intestinal FXR signaling (111). Specifically, FXR inhibition enhanced gluconeogenesis and glycogenolysis in the liver and increased insulin sensitivity in fat and skeletal muscle (108), as evidenced by enhanced lipolysis and decreased triglyceride accumulation, in agreement with the results observed in the experiment (111).

Thirdly, gut microbes are instrumental in maintaining the integrity of the intestinal barrier function. Loss of intestinal barrier integrity raises the exposure of the liver to bacterial pro-inflammatory products (e.g., LPS) and toxic bacterial metabolic by-products, and this could be another possible hypothesis for the impact of intestinal microbes on MAFLD and NASH (112). Li et al. implemented a dietary intervention experiment in rats and showed that intestinal mucosal barrier dysfunction may be an important contributing factor in NASH rats (113).

Finally, fermentation of carbohydrates by intestinal bacteria leads to the production of endogenous ethanol which could promote MAFLD (87). Mezey et al. identified ethanol in the blood of morbidly obese patients (114). Similarly, Cope et al. detected ethanol in the exhaled gas of obese mice with no alcohol intake (115). Children with NASH have elevated blood ethanol concentrations compared to healthy individuals or children with MAFLD, suggesting that endogenous ethanol production may contribute to worsening liver damage by stimulating inflammatory signals (87).

### The Intestine-Liver Axis Is the Structural Basis for the Interaction of Intestinal Microorganisms With MAFLD

The bidirectional exchange of substances between the intestine and the liver cannot be achieved without the intestine-liver axis (11), a communication pathway that realizes its anatomical and functional bidirectional action in the intestine and liver mainly through the portal vein and bile ducts (116, 117). In this process, the liver secretes nutrients such as BAs and antibodies and other biologically active substances through the bile duct to the upper part of the small intestine, where these components travel down the intestinal tract. The portal vein absorbs endogenous BAs and other substances of exogenous origin from the metabolism of the gut (and microorganisms in the gut) into the blood for transport to the liver (11). For instance, on the one hand, the metabolite bile salts utilize nuclear receptors (e.g. FXR, TGR5) as essential signaling molecules to regulate hepatic bile acid synthesis, glucose metabolism, lipid metabolism and energy utilization in the diet (11), affecting the composition of the intestinal flora and the integrity of the intestinal barrier (118). On the other hand, intestinal factors conversely influence bile acid synthesis, glucose and lipid metabolism in the liver (119). It has been suggested that intestinal flora can have a profound effect on BA metabolism by promoting uncoupling, dehydrogenation and dehydroxylation of primary BAs in the distal small intestine and colon, leading to improved chemical diversity of BAs and involvement in the gut-liver axis (119).

## Dietary Nutrients Affect MAFLD Through Intestinal Microenvironment and Microbiota

**Figure 2:**
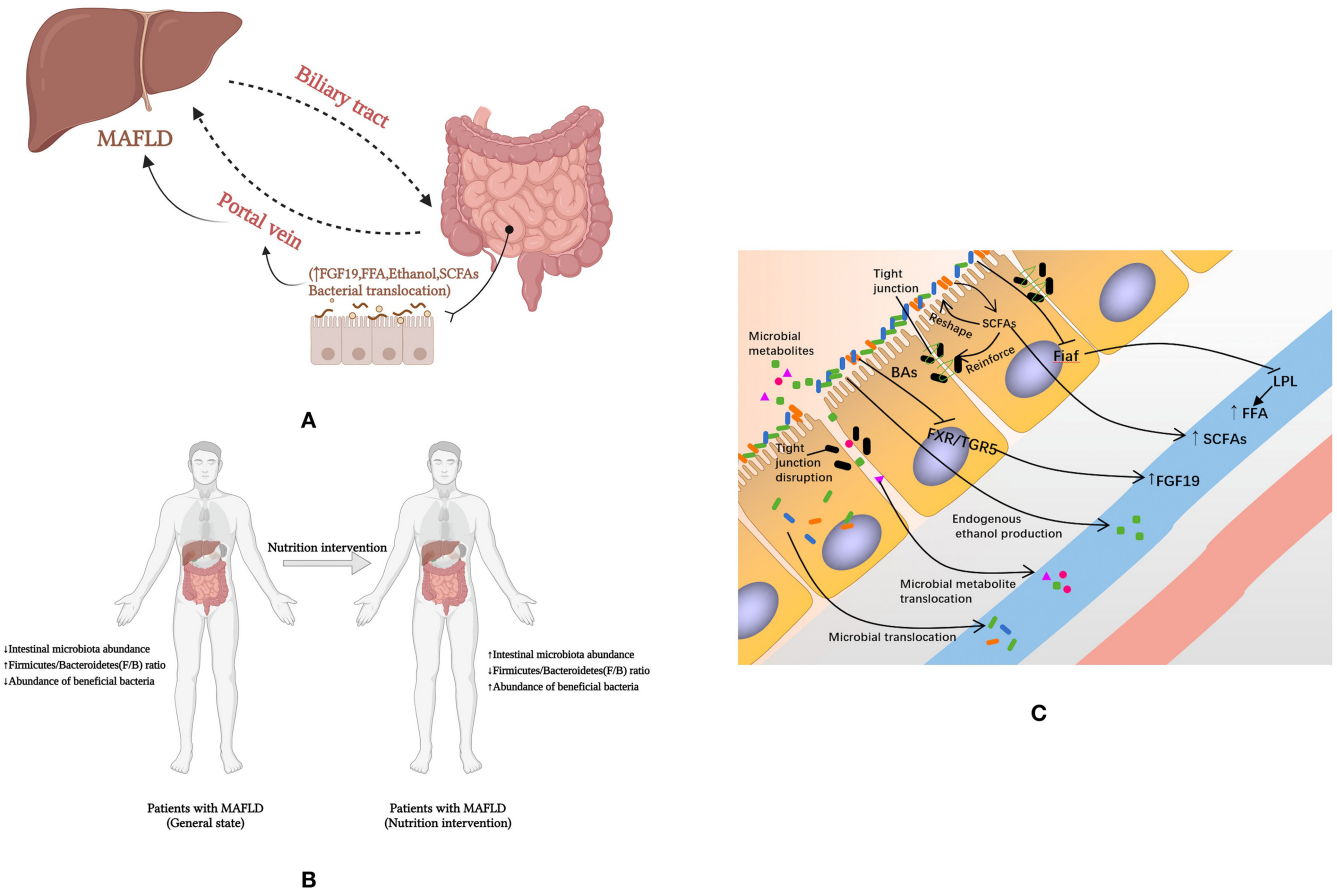
How nutrients affect MAFLD through intestinal microenvironment and microbiota. **(A)** Intestinal microbial-intestinal-liver axis; **(B)** Macro level evidence on the effect of nutrients on MAFLD; **(C)** The mechanisms by which nutrients affect MAFLD through the intestinal microenvironment and microflora. FFA, free fatty acids; Fiaf, fasting-induced adipocyte factor; LPL, lipoprotein lipase; MAFLD, fatty liver disease associated with metabolic dysfunction; SCFA, short-chain fatty acid; FXR, farnesol X receptor; TGR5, Takeda G protein-coupled receptor 5; BAs, bile acids; FGF 19, fibroblast growth factor 19.

Dietary nutrients enter the digestive tract and are digested and absorbed, with the liver and intestines being the key digestive organs. The liver and the intestine communicate in both directions through the portal vein, the bile duct and the body circulation. Dietary nutrients such as BAs and amino acids are metabolized endogenously by intestinal tract (and microbes in intestinal tract) into blood, and then transported to liver (11). The presence of the intestinal microbial-intestinal-liver axis is an essential physiological basis for nutrients to influence the development of MAFLD through gut microbes (Figure 2A). However, few studies have focused on the effects of dietary nutrients on the development of MAFLD by affecting the intestinal microflora. Therefore, we present all the studies that conducted dietary nutrients as interventions and documented the changes in gut microbiota and metabolic parameters in MAFLD animals or patients in Supplementary Table S1 (120–131). Most of these studies are limited to the intervention of fiber. In addition, we sought additional evidence to elucidate the effects of dietary nutrients on MAFLD through intestinal microbiota and the intestinal microenvironment.

### Dietary Nutrients May Reverse Intestinal Disorders in Patients With MAFLD

Gut microbial disorders in patients with MAFLD are characterized by a low intestinal microbiota abundance (96), an increased *F/B* ratio (132), and a reduced abundance of beneficial bacteria (96, 101, 102) to reestablish the balance of gut microbes. However, dietary interventions may have the opposite benefit on the gut microbiota (Figure 2B).

#### Enrich Intestinal Microbiota Abundance

Carbohydrates can alter the composition and function of gut microbes (133, 134). Some animal experiments have shown that fructose intake is responsible for altering intestinal microbiota, mucosal status, and liver homeostasis in mice. The main manifestations were a decrease in the ratio of the thick-walled phylum/Bacillus phylum (69). In a dietary intervention study, the relative abundance of *Bacillus/Plasmodium spp*. was changed in male rats subjected to a high-fat, high-sucrose diet intervention (135). The relative abundance of a variety of intestinal microorganisms reduced at different time points in the intervention group of rats compared to the control group (135).

The effect of lipids on the gut microbiota is significant, with high-fat diets often exhibiting increased gut microbiota abundance compared to low-fat diets (36, 136). A three-week randomized crossover study assessed the beneficial effects of almond(fat-rich food) consumption on the composition of the gut microbiota. Eighteen healthy subjects were randomized to consume food containing almonds and food not containing almonds, and a comparison after 3 weeks of intervention revealed that almond consumption had an effect on the relative abundance of microorganisms in the gastrointestinal tract, as demonstrated by an increase in the relative abundance of *Lachnospira, Roseburia and Dialister* with almond consumption (137). Both the milkfat and corn oil diets resulted in increased alpha diversity, with species richness and Chao1 increasing with corn oil and milkfat exposure, whereas the olive oil diet resulted in similar richness to low-fat foods. Dietary lipid types conferred differences in the core functions of each microbial community (36).

Amino acids of dietary or endogenous origin can be utilized by intestinal microorganisms for protein synthesis (138), and to provide metabolic energy to the intestinal flora (139). Changes in dietary protein content may lead to changes in the composition and function of the gut microbiota. In a calorie-controlled dietary intervention trial in which 80 overweight and obese subjects were randomized to either a high-protein or a normal-protein diet, the alpha diversity of the intestinal flora remained unchanged from baseline in the normal-protein group after 8 weeks, whereas it increased significantly in the high-protein group. The study noted that the high-protein diet intervention resulted in differences in the abundance of genera compared to the normal-protein diet group (140). Many studies have also shown that soy protein is more closely associated with the diversity of gut microbes than milk protein (52, 141–143).

A previous report showed that the composition of the gut microbiota in mice affects host selenium levels and the expression of selenoproteins in the host (144). On the other hand, selenium levels have also been shown to enhance overall microbiota diversity in mice (145) increased intestinal microbial diversity in adult male rats following ingestion of a magnesium-rich marine mineral mixture (146).

An association between vitamin A and gut microbes is potentially possible. Hibberd and colleagues investigated the effects of different micronutrients (vitamin A, folic acid, iron and zinc) on the regulation of intestinal microbiology and found that vitamin A deficiency had the greatest impact on gut microbes metabolism (147). It has been found that the microbial communities of vitamin A-enriched children are more diverse than those of vitamin A-deficient children (148).

It was found that the intestinal microbiota of subjects who drank coffee daily presented significantly higher relative abundance of synergistic flora compared to subjects who drank coffee regularly (*P* = 0.01, FDR = 0.10) or not at all (*P* = 0.01, FDR = 0.08) (149).

#### Reduce F/B Ratio

Mice fed with fructose syrup for 12 weeks showed a rise in the genera *Coprococcus* and *Ruminococcus* of *Firmicutes* compared to mice fed a standard diet with water (69). A recent animal study intervened in 32 three-week-old C57BL/6 mice on a high-fat and low-fat diet, with the high-fat derived from olive oil, corn oil or anhydrous milk fat (36). Studies have shown that olive oil high-fat diets are associated with intestinal oxygen-tolerant microorganisms and lead to increased abundance of various thick-walled phyla such as *Clostridiaceae* (*P* = 0.003) and *Streptococcaceae* digestiveis (*P* = 0.01). Corn oil, in turn, increased the abundance of members of the genus *Clostridium* faecalis and the *Firmicutes* family from the *Turicibacteraceae (P* = 0.008) (36). Dairy fat promoted different families of thick-walled bacteria, including *Bacillus tansy* (*P* = 0.008) and several genera of *ruminal cocci* (*P* = 0.003) (36). Animal experiments found that coffee reduced the percentage of the thick-walled phylum *Bacillus/bacteroidetes* in the intervention group after 10 weeks compared to the control group without caffeine or coffee in the water (150, 151).

#### Promote Abundance of Beneficial Gut Microbes to Reestablish the Balance of Gut Microbes

Probiotics have been widely studied for their protective effects on intestinal and host health, particularly *Lactobacillus* and *Bifidobacterium* (152). The protective effects of probiotic intestines may be attributed to their ability to resist harmful substances, lower intestinal pH value, reduce colonization by other microorganisms and repair the intestinal barrier (26, 153). These microorganisms reestablish the balance of gut microbes by lowering the intestinal pH and competing with pathogenic microbes for survival (154). Certain probiotics have been shown to improve intestinal barrier function by restoring mucus layer thickness, strengthening TJs proteins and producing specific antimicrobial and bioactive lipids with anti-inflammatory properties (155). On the other hand, probiotics also produce SCFAs that affect intestinal barrier function by activating GPR-41 and GPR-43, which are expressed on enteroendocrine L cells and promote the secretion of intestinal peptides (GLP-2) (156, 157).

There are numbers of fiber and carbohydrates that increase the abundance of prebiotics (158). Prebiotics include oligosaccharides, inulin, fructooligosaccharides and isomalto-oligosaccharides, which cause specific changes in the composition and/or activity of the gastrointestinal microbiota, are degraded by bacterial enzymes in the intestine and inhibit the growth of pathogenic bacteria by producing bacteriocins and SCFAs, thereby promoting the growth of probiotics (159). A comparable research by liu et al. found significant increases in *Bifidobacterium spp*. and *Lactobacillus spp*. in the almond and almond peel groups, moderate changes in *E. coli* populations and significant inhibition of *Clostridium perfringens* growth in both almond intervention groups compared to the control group (92). In addition to this, many studies have reported that nuts such as walnuts and other plant-derived fats such as soybean oil increased *Bifidobacterium spp., Rhodobacter spp. and Bacillus faecalis spp*. showing biological benefits and protective properties (160). An animal study used saturated and unsaturated fatty acids as fat sources for a high-fat diet and a low-fat diet intervention in C57BL/6 N mice, respectively. This study showed that mice fed on soybean oil (high in polyunsaturated fat) exhibited lower relative abundance of *Aspergillus, Clostridium perfringens, Heterobacterium, and Deltaproteobacteria* compared to mice fed on coconut oil (high-saturated fat).

One hundred and thirty-nine Ivorian children who received iron-fortified biscuits (Fe:20 mg/d) for 6 months presented with reduced abundance of intestinal microbiota, increased abundance of harmful bacteria and reduced abundance of Lactobacilli (161). However, an interventional study of iron in healthy infants discovered that consumption of high iron formulas was linked to a significant reduction in the abundance of Bifidobacteria compared to low iron formulas, while no enhanced growth of pathogenic bacteria was detected (162). The impact of iron on the intestinal flora is influenced by the chemical form of dietary iron (163), the dose (162, 164, 165) and the mode of administration (162, 166). Animal experiments with zinc supplementation (120 mg/kg) in the “Salmonella typhimurium attack” model have suggested that zinc supplementation regulates the cecum by enhancing the number of total bacteria and beneficial Lactobacillus bacteria and reducing the number of Salmonella microbiota (167). Shen et al. used the same dose of iodine (18 μg/kg/day) for 8 weeks in obese mice on a high-fat diet and normal mice on a normal-fat diet (168). It was observed that the elevated thyroid hormone concentrations in the obese mouse model were accompanied by dysbiosis of the intestinal flora, as evidenced by an increased abundance of harmful bacteria and a decrease in beneficial bacteria, such as Fecalibacterium prausnizii, which is associated with butyrate production (168). However, in normal mice, iodine had a beneficial effect on the intestinal microbiota by increasing the levels of Bifidobacterium, Lactobacillus, Fecalibacterium and Allobaculuum (168). It has been reported that magnesium deficiency for 4 days reduces the level of Bifidobacteria in the cecum of mice, but with longer duration of magnesium deficiency (3 weeks), the level of Bifidobacteria and Lactobacilli in the intestine improves (169).

Clinical trials have provided extensive evidence that a high-fiber diet can decreases the *F/B* ratio and increases the abundance of beneficial microflora, including mainly *Bifidobacterium* (170–172), *Lactobacillus* (172), *Bacillus* (173, 174) and *Prevotella* (175, 176). Similar conclusions have been drawn from animal experiments. Intestinal *Bifidobacterium spp., Lactobacillus spp*. and *Rhodobacter spp*. increased in male obese rats after a continuous 6-week intervention with 10% oligofructose. A study showed that inulin and oligofructose diets stimulated the growth of *Lactobacillus* intestinalis in rats (177).

Rodent studies demonstrate that vitamin D deficiency by dietary restriction, lack of CYP27B1, or lack of VDR promote increases in the *Bacteriodetes* (178–181) and *Proteobacteria* (178–180). In a cross-sectional study involving 98 healthy individuals, vitamin D intake was found to be negatively associated with the abundance of *Prevotella* and strongly positively associated with those of *Bacteroides* (182). However, some researchers reached different conclusions, presumably because the results were influenced by methodological differences in vitamin D “doses” (e.g., sunlight exposure, diet and nutrient supplementation) (165, 183, 184).

A human intervention study showed that consumption of green tea for 10 days improved the proportion of *Bifidobacteria* (185). Another study found that 1,000 ml of green tea per day is associated with an increase in *Bifidobacteria* and improved colon bacterial characteristics (186). Animal experiments have demonstrated something similar. With the intervention of Gampo Tea (GTE, an emerging tea beverage produced from the peels of pu-erh tea and citrus), well known probiotics such as *Bifidobacterium, Lactobacillus* and *Lactococcus* were enriched in the intestinal flora of rats in the GTE group (187). The possible mechanism for the inhibitory effect of tea phenolics on intestinal flora is the ability of tea phenolics to disrupt cell membranes (188). For Gram-positive bacteria, EGCG directly binds and disrupts their exposed peptidoglycan layer, leading to reduced protection and bacterial inactivation. In contrast, Gram-negative bacteria are not significantly affected by EGCG due to an additional outer membrane protection that blocks the damaging effect of EGCG. In addition, the outer membrane of Gram-negative bacteria consists of lipopolysaccharides (189), which are negatively charged even in a pH-neutral environment, and this could repel the proximity of EGCG (189). The increased content of bacilli (186, 190) suggests that some Gram-positive bacteria seem to be insensitive to catechins. In addition, Hidetoshi et al. showed that catechins can react with dissolved oxygen in aqueous solutions to produce hydrogen peroxide (191), further damaging intestinal flora cell surface proteins and triggering endogenous oxidative stress (192).

Of interest is that because it contains chlorogenic acid to acidify intestinal pH value, coffee seems to have an anti-harmful flora effect (193, 194). Coffee increases the number and/or activity of *Bifidobacterium, Lactobacillus* (90) and decreases the amount of *Clostridium* and *Escherichia* coli (90, 91), while the effect on the number of Enterococcus was not significant (90). The results of the antibacterial activity assay showed that coffee inhibited the growth of *E. coli* and *Enterococcus faecalis*. However, one report stated that 3 cups of coffee per day for 3 weeks increased *Bifidobacterium spp*. without any effect on other dominant microbiota bacteria.

### The Potential Mechanisms by Which Dietary Nutrients Affect MAFLD Through the Intestinal Microenvironment and Microflora

Diet produces a variety of products through bacterial metabolism. SCFAs reduce the pH of the intestinal microenvironment and help maintain or reshape healthy intestinal microbial homeostasis, as well as enhance the stability of TJs. SCFA that are transferred to the body cycle may directly affect intestinal physiology and motility by binding to G protein-coupled receptors (GPRs) and participate in host metabolism (195, 196). These receptors were found to be expressed in several metabolically active tissues and are involved in the response and regulation of many processes, including glucose homeostasis and lipid metabolism (197, 198). Evidence also suggests that SCFAs may act as histone deacetylase inhibitors to regulate gene expression (199), and dietary SCFA intake improves hepatic metabolic conditions via FFAR3 signaling pathway (200). SCFAs contribute to the remodeling of TJ. As mentioned earlier, butyric acid enema helps reduce intestinal inflammation in IBD patients (57), preserving TJ integrity and directly improving intestinal barrier function (58–60). In addition, gene expression of Fiaf in intestinal epithelial cells can be inhibited by intestinal microflora increasing plasma FFA levels by LPL.

BAs bind to FXR/TGR5 bile acid receptors. The nuclear receptor FXR is a transcriptional regulator (201) that is involved in both the regulation of glucose, lipid and energy homeostasis and is also essential for regulating the homeostatic negative feedback loop of BA synthesis and distribution. BAs activate FXR to produce antimicrobial peptides such as human β defensin-1 and 2, which inhibit the overgrowth of intestinal microbiota and are responsible for maintaining intestinal mucosal barrier function and regulating inflammation (202, 203). FXR/TGR5 inhibition enhanced hepatic gluconeogenesis and glycogenolysis and increased insulin sensitivity in fat and skeletal muscle, while activated FXR further facilitates that synthesis of fibroblast growth factor 19.

Intestinal bacteria break down carbohydrates to endogenous ethanol, which is metabolized in the liver by enzyme ethanol dehydrogenase. Ethanol is thought to be an important factor contributing to MAFLD. On the one hand, excess ethanol leads to fluctuations in intracellular redox potential changes and leads to an increased inflammatory response (204, 205). On the other hand, ethanol metabolism produces obligatory redox changes that promote the accumulation of triglycerides in hepatocytes, increase portal blood ethanol levels and induce hepatic steatosis. The possibility that a slight increase in ethanol exposure facilitated the series of events that eventually led to hepatic steatosis remains plausible as found in the mouse experiments performed by Cope et al. (115). In addition, high levels of ethanol-producing bacterial strains in the gut [e.g., *Escherichia coli, Bacteroides* and *Clostridium* (87–89)] can also accelerate the development of MAFLD (88).

Intestinal permeability may have a role in the pathogenesis of metabolism-related diseases such as MAFLD. Increased intestinal permeability has been found to be associated with increased levels of endotoxin in patients with MAFLD, and correlates with liver disease severity and levels of TJ destruction (206, 207). It is believed that changes in intestinal permeability significantly affect metabolism because the intestinal barrier plays a key role in the transport of nutrients and macromolecules, while providing an effective barrier to harmful macromolecules and microorganisms (208). At the same time, the loose TJs allow nutrients and other microbial material to cross the intestinal epithelium and target white adipose tissue, thereby increasing adipokine production (leptin and resistin), adipocyte size and SVF cell numbers (209–212). Dietary habits may be associated with the initial intestinal barrier defect through direct disruptive effects of food agents, secondary dysregulation due to low-grade inflammation or specific changes in the microbial composition affecting the barrier, which is the theoretical basis for interventions in MAFLD using substances such as prebiotics (213, 214). The potential mechanisms by which dietary nutrients affect MAFLD through the intestinal microenvironment and microflora were showed in Figure 2C.

## Conclusion

**Figure 3:**
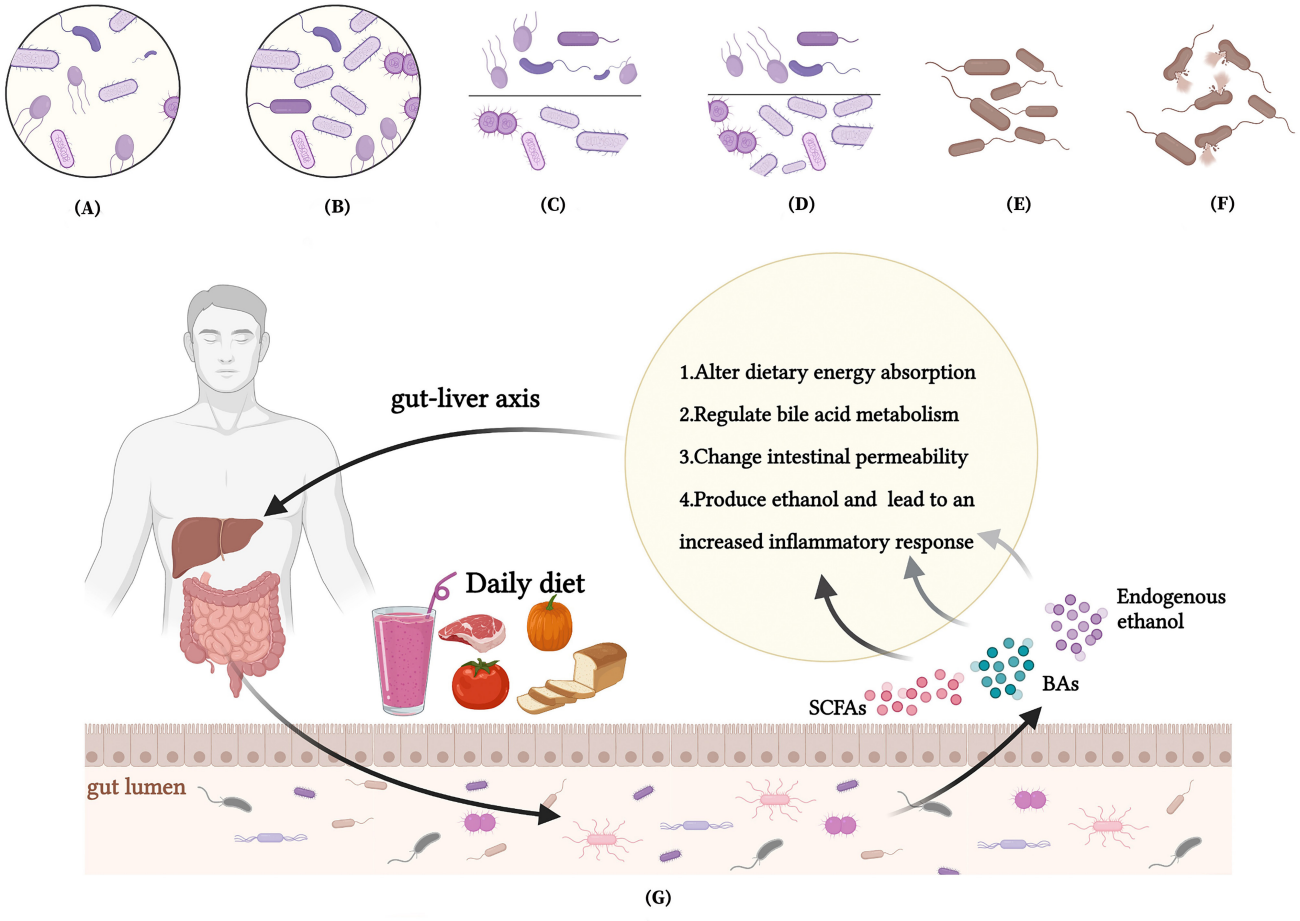
Interactions between nutrients, intestinal microbiota and MAFLD. **(A)** A low intestinal microbiota abundance in MAFLD patients; **(B)** Dietary interventions enrich the intestinal microbiota abundance of MAFLD patients; **(C)** An increased *Firmicutes/Bacteroidetes(F/B)* ratio in MAFLD patients; **(D)** Dietary interventions reduce F/B ratio of patients with MAFLD; **(E)** A reduced abundance of beneficial bacteria in MAFLD patients; **(F)** Dietary interventions promote abundance of beneficial gut microbes to reestablish the balance of gut microbes; **(G)** How nutrients affect MAFLD through intestinal microenvironment and microbiota.

The maintenance of human life and health is dependent on the support of dietary nutrients. Our review suggests that there is an interaction between the dietary nutrients, gut microbiota and MAFLD. These dietary nutrients influence the development and progression of MAFLD mainly through the hepatic-intestinal axis by altering dietary energy absorption, regulating BAs metabolism, changing intestinal permeability and producing ethanol. Meanwhile, the nutrients have the ability to combat MAFLD in terms of enriching abundance of intestinal microbiota, reducing *F/B* ratio and promoting abundance of beneficial gut microbes (Figure 3). Therefore, family therapy with MAFLD using a reasonable diet could be considered.

## Author Contributions

NY, YY, XL, and YW designed the research. NY, JL, XW, and RG conducted the research. NY, YY, ZX, and XL wrote the paper. NY had primary responsibility for final content. All authors read and agreed with the final manuscript.

## Conflict of Interest

The authors declare that the research was conducted in the absence of any commercial or financial relationships that could be construed as a potential conflict of interest.

## Publisher's Note

All claims expressed in this article are solely those of the authors and do not necessarily represent those of their affiliated organizations, or those of the publisher, the editors and the reviewers. Any product that may be evaluated in this article, or claim that may be made by its manufacturer, is not guaranteed or endorsed by the publisher.
