# Supplementary material for: Effects of Dietary Nutrients on Fatty Liver Disease Associated With Metabolic Dysfunction (MAFLD): Based on the Intestinal-Hepatic Axis
Source: Front Nutr. 2022 Jun 17;9:906511. doi: 10.3389/fnut.2022.906511 (PMC9247350; doi:10.3389/fnut.2022.906511)
Supplement: Supplementary file 1 [file Table_1.DOCX]

| **Table S1: Nutrition, gut microbiota, and metabolic regulation with MAFLD.** | | | | | | |
| --- | --- | --- | --- | --- | --- | --- |
| **Nutrition** | **Study** | **Subject Characteristics** | **Study Design** | **Intervention** | **Changes Related to Gut Microbiota** | **Changes Related to Metabolic Regulation** |
| Fiber | Yvonne Ritze et.al, PLoS One(117) | C57bl6/J mice: animal models of NAFLD  n=8 (Control) n=8(High-fat diet+arabinoxylans, HF+AX) n=8(High-fat diet,HF) | 4weeks | (1)Control group((AO4, SAFE, Villemoison-sur-Orge, France) (2)HF group(fat for 60%, carbohydrate for 20% and protein for 20%) (3)HF+AX group(90% HF (w/w)+10%AX) | **HF+AX vs. HF:** ↑ *Roseburia spp, Bacteroides-Prevotella spp, Bifidobacterium spp* ,expression of tight-junction proteins, proglucagon | **HF+AX vs. HF:** ↑ZO-1, occludin,insulin resistance index ↓adiponectin,LDL,HDL |
| Fiber | Marc R Bomhof et al, Obesity (Silver Spring)(118) | Sprague-Dawley rats:animal models of NAFLD  n=10 (Control) n=10(Oligofructose, OFS) n=10(B. animalis subsp. lactis BB-12, BB-12) n=10(OFS + BB-12) | 8weeks | (1)Control group(AIN-93M diet) (2)OFS group(10% (wt/wt),Orafti P95, BENEO-Orafti Inc.) (3)BB-12(1 × 1010 CFU/d) (4)OFS + BB-12(10%OFS + BB-12) | **OFS vs. control group** ↑*Bacteroides spp.* ↑*Lactobacillus spp.* ↑*Bifidobacterium spp.* ↑*B. animalis*  ↓*C. coccoides* ↓*C. leptum, Clostridium Cluster XI and I* ↓*Enterobacteriaceae* | **OFS vs. control group** ↑GPL-1 ↑PYY(peptide tyrosine tyrosine) AUC ↓Glucose |

Significant results are indicated by an up/down arrow.

| **Table S1: Nutrition, gut microbiota, and metabolic regulation with MAFLD. (Continued)** | | | | | | | |
| --- | --- | --- | --- | --- | --- | --- | --- |
| **Nutrition** | **Study** | **Subject Characteristics** | **Study Design** | **Intervention** | | **Changes Related to Gut Microbiota** | **Changes Related to Metabolic Regulation** |
| Fiber | Barbara D Pachikian et.al, Mol Nutr Food Res.(119) | Male C57Bl/6J mice：animal models of NAFLD  n=8(n-3 PUFA-depleted diet /fructo-oligosaccharides ,DEF/FOS) n=7(DEF) n=4(Control) | last 10days | (1)DEF/FOS( an n-3 PUFA-depleted diet+0.25 g of FOS/day/mice) (2)DEF( an n-3 PUFA-depleted diet) (3)Control | | **DEF/FOS vs. DEF** ↑*Bifidobacterium spp.* ↑total bacteria count  ↓*Roseburia spp.* | **DEF/FOS vs. DEF** ↓TG  ↓free cholesterol content ↓cholesterolemia ↓LDLc  ↓HDLc ↓SREBP-2 |
| Fiber | Audrey M. Neyrinck et.al, PLoS One.(120) | male C57bl6/J mice：animal models of NAFLD  n=8(Control) n=8(High-fat diet,HF) n=8(water-extractable high molecular weight arabinoxylans+HF, AX+HF) | 4 weeks | (1)Control group(AO4, SAFE, Villemoison-sur-Orge, France) (2)HF group(fat for 60%, carbohydrate for 20% and protein for 20%) (3)HF+AX group(90% HF (w/w)+10%AX) | | **HF+AX vs. HF:** ↑*Bacteroides–Prevotella spp.*  ↑*Roseburia spp.*  ↑*bifidobacteria* ↑*bifidobacterium animalis ssp lactis* | **HF+AX vs. HF:** ↓adiponectin, HDL,LDL ↑insulin resistance index ↑the shift from hepatic free cholesterol towards esterified cholesterol |
| Significant results are indicated by an up/down arrow. | | | | |  |  |  |

| **Table S1: Nutrition, gut microbiota, and metabolic regulation with MAFLD. (Continued)** | | | | | | | | |
| --- | --- | --- | --- | --- | --- | --- | --- | --- |
| **Nutrition** | **Study** | **Subject Characteristics** | **Study Design** | **Intervention** | | **Changes Related to Gut Microbiota** | | **Changes Related to Metabolic Regulation** |
| Fiber | P D Cani et.al, Gut.(121) | obese C57BL/6 mice:  n=10(Ob-CT,Control) n=10(Ob-Pre, fermentable dietary fibre, oligofructose)  n=10(Ob-Cell, non-fermentable dietary fibre (microcrystalline cellulose) | 5 weeks | (1)Ob-CT(A04, Villemoisson sur Orge) (2)Ob-Pre(Orafti, Tienen) (3)Ob-cell(Vivapur Microcrystalline cellulose) | | **Ob-Pre vs. Ob-CT:** ↑total bacteria count ↑*Lactobacillus spp.* ↑*Bifidobacterium spp.* ↑*C coccoides–E rectale cluster* **Ob-Pre vs. Ob-Cell:** ↑total bacteria count ↑*Lactobacillus spp.* ↑*Bifidobacterium spp.* ↑*C coccoides–E rectale cluster* | | **Ob-Pre vs. Ob-CT:** ↑GLP-1 ,↑GLP-2, ↑ZO-1 and occludin, ↓glucose-dependent insulinotropic polypeptides (GIPs), LPS,PAI-1,NADPHox,iNOS,TLR4,TNF-α ↓hepatic expression of inflammatory and oxidative stress markers |
| Fiber | Aafke W. F. Janssen et.al, J Lipid Res.(122) | C57bl6/J mice：animal models of NAFLD  n=not mentioned (Control group, CTRL) n=not mentioned (resistant starch, RS group) n=not mentioned (guar gum, GG group) | 18 weeks | (1)Control(formula D12450B or D12451 from Research Diets ,no dietary fiber) (2)RS group (fermentable dietary fiber, resistant starch,brand name C*Actistar 11700) (3)GG group(brand name Viscogum) | | **GG vs. RS group** ↑ *Bifidobacterium* ↑ *Prevotella* ↑ *Akkermansia* ↓ *Oscillospira* ↓ *SMB 5 3 (family Clostridiaceae)* | | **GG vs. RS group** ↓hepatic steatosis, ethanol ↑hepatic inflammation ↑hepatic fibrosis ↑SCFAs **GG vs. CTRL group** ↓bodyweight ↑body glucose tolerance ↓fasting plasma insulin levels |
| Fiber | De Faria Ghetti et al., J Gastrointestin Liver Dis, 2019(123) | n = 40, NASH, BMI 31, 50.6 y (Control), 48.3 year (DIET), M/F | 3 months Parallel | (1) Control group (nutritional orientation) (2) The DIET group (fiber 30 g/day + nutritional orientation) | | **Within groups:** ↑Density of total microorganisms ↓*Bacteroidetes, Verrucomicrobiales* | | ↓Ins, HOMA-IR, TC **Within groups:** ↓Glu, HOMA-IR, TC, TG ↓TC, LDL-C, TG |
| Significant results are indicated by an up/down arrow. | | | | |  | |  |  |

| **Table S1: Nutrition, gut microbiota, and metabolic regulation with MAFLD. (Continued)** | | | | | | |
| --- | --- | --- | --- | --- | --- | --- |
| **Nutrition** | **Study** | **Subject Characteristics** | **Study Design** | **Intervention** | **Changes Related to Gut Microbiota** | **Changes Related to Metabolic Regulation** |
| Fiber | Marc R Bomhof et.al, Eur J Nutr.(124) | NAFLD,n=8(prebiotic,PRE) n=6(isocaloric maltodextrin placebo, PLA) BMI:33.7 ± 3.0 kg/m 2(PRE), 34.8 ± 2.2kg/m 2(PLA) Age:45.3 ± 5.6 years(PRE), 53.3 ± 4.8 years(PLA)M/F | 36 weeks Parallel | (1)PRE group ( oligofructose, 8 g orally per day for 36 weeks) (2)PLA group (oligofructose, 8 g orally per day for 12 weeks+ 16 g per day for 24 weeks or an isocaloric maltodextrin placebo) | **Between groups:**  ↑*Bifidobacterium spp.*  ↑*Actinobacteria* | **Within groups:(PRE)** ↓IL-6,TNF-α,without significantly difference ↓total NAS **Between groups:** ↓hepatic steatosis |
| Carbohydrate(polysaccharide) | Charalampia Amerikanou et.al, Mol Nutr Food Res.(125) | NAFLD/NASH, n=41(Mastiha) n=57(placebo) Age: 18–67 years BMI >30 kg m-2 | 6 months Parallel | (1)Mastiha group(2.1g/day) (2)Control group( corn starch,2.1g/day) | **Between groups:** ↓Bray-Curtis dissimilarity index ↑*Fusobacterium*  ↑*Prevotella 9* | **Between groups:** ↓cholic acid, Lysophosphatidylcholines, Lysophosphatidylethanolamines |
| Carbohydrate(wild rice) | Xiao-Dong Hou et.al, Int J Mol Sci.(126) | C57bl6/J mice: animal models of NAFLD  n=12(HFD+wild rice) | 11weeks parallel | (1) High-fat diet group, HFD(normal chow containing 10% lard (2) Wild rice diet groups (normal chow+10% lard + 10% wild rice or 20% wild rice) | **Within groups:**  ↓Bacteroidetes, Prevotella, Bacteroides, and Staphylococcus  ↑Firmicutes, Lactobacillus | **Within group:**  ↓low-grade inflammation  ↑NF-κB |
| Significant results are indicated by an up/down arrow. | | | | | | |

| **Table S1: Nutrition, gut microbiota, and metabolic regulation with MAFLD. (Continued)** | | | | | | |
| --- | --- | --- | --- | --- | --- | --- |
| **Nutrition** | **Study** | **Subject Characteristics** | **Study Design** | **Intervention** | **Changes Related to Gut Microbiota** | **Changes Related to Metabolic Regulation** |
| green tea | Ulrika Axling et.al,Nutr Metab (Lond).(127) | C57BL/6J mice:animal models of NAFLD  n=21(Control)  n=21(Green Tea) | 22 weeks Parallel | (1) Control group (high-fat diet) (2) The GT group (green tea powder + high-fat diet) | **GT vs. control group**  ↑ Shannon (H') and Simpsons’s diversity index | **GT vs. control group** ↓ fasting plasma glucose, insulin, fructosamine, HDL receptor SR-B1 **↓** HOMA-IR,ALT, TC, LDL receptor |
| Fiber, Protein | Dolan C Saha et.al, Nutr Res.(128) | Wistar rats：animal models of NAFLD  n=8-10 (Control) n=8-10(High fiber,HF) n=8-10(High protein,HP) | 4 weeks | (1)Control group(AIN-93G purified diet) (2)HF group(3.30 kcal/g ,high fiber diet) (3)HP group(3.76 kcal/g, high protein diet) | **HF vs. control group:** ↑Total bacteria ↑*Bifidobacteria* ↑*Bacteroides:Prevotella* **HF vs. HP:** ↓*Firmicutes,Firmicutes:Bacteroidetes,Bacteroides:Prevotella* | **HF vs. control group:** ↓TG, Leptin mRNA level  ↑3-hydroxy-3 methylglutaryl-CoA (HMG-CoA),Cholesterol 7-α-hydroxylase (CYP) mRNA levels **HF vs. HP** ↓TG,Leptin mRNA level, hepatic cholesterol content Negative correlation between liver weight **HP vs. control group:** ↑ Leptin mRNA level,3-hydroxy-3 methylglutaryl-CoA (HMG-CoA) |
| Significant results are indicated by an up/down arrow. | | | | | | |
